# Comparative Effectiveness of Adjuvant XELOX Versus TS-1 Monotherapy After D2 Gastrectomy for Stage III Gastric Cancer: A Real-World Nationwide Cohort Study

**DOI:** 10.3390/life16071069

**Published:** 2026-06-26

**Authors:** Meng-Hsing Ho, Chih-Wei Yang, Po-Huang Chen, Jia-Hong Chen, Ping-Hsuan Hsieh, Heng-Jun Lin, Li-Yuan Bai, Cheng-Hsiang Lo, Yu-Guang Chen, Ching-Liang Ho

**Affiliations:** 1 Division of General Surgery, Department of Surgery, Tri-Service General Hospital, National Defense Medical University, Taipei 114202, Taiwan; hmh720208@gmail.com; 2 Division of Gastroenterology, Department of Internal Medicine, Tri-Service General Hospital, National Defense Medical University, Taipei 114202, Taiwan; youngwayleon@gmail.com; 3 Division of Hematology/Oncology, Department of Internal Medicine, Tri-Service General Hospital, National Defense Medical University, Taipei 114202, Taiwan; chenpohuang@hotmail.com (P.-H.C.); ndmc_tw.tw@yahoo.com.tw (J.-H.C.); 4 College of Pharmacy, National Defense Medical University, Taipei 114202, Taiwan; pinghsuan.h@mail.ndmutsgh.edu.tw; 5 Management Office for Health Data, China Medical University Hospital, China Medical University, Taichung 404327, Taiwan; 038958@tool.caaumed.org.tw; 6 Division of Hematology and Oncology, China Medical University Hospital, China Medical University, Taichung 404327, Taiwan; 013664@tool.caaumed.org.tw; 7 Department of Radiation Oncology, Tri-Service General Hospital, National Defense Medical University, Taipei 114202, Taiwan; lsir183@yahoo.com.tw; 8 The Center for Cell Therapy and Regenerative Medicine, Tri-Service General Hospital, Taipei 114202, Taiwan; 9 Graduate Institute of Life Sciences, National Defense Medical University, Taipei 114202, Taiwan; 10 Division of Hematology/Oncology, Department of Internal Medicine, Taipei Tzu Chi Hospital, Buddhist Tzu Chi Medical Foundation, New Taipei 231016, Taiwan

**Keywords:** gastric cancer, adjuvant chemotherapy, TS-1, S-1, XELOX, CAPOX, D2 gastrectomy, propensity score matching, overall survival, real-world evidence

## Abstract

Adjuvant XELOX (capecitabine plus oxaliplatin) and TS-1 (S-1) monotherapy are both guideline-recommended following D2 gastrectomy for gastric cancer, yet head-to-head real-world data exclusively in stage III disease remain scarce. Using Taiwan’s National Health Insurance Research Database linked to the Taiwan Cancer Registry–Long Form, we identified stage III gastric cancer patients who underwent D2 gastrectomy (2010–2019) and received adjuvant XELOX or TS-1 for ≥3 months. Propensity score matching balanced chemotherapy and non-chemotherapy cohorts (1706/group). Overall survival (OS) was the primary endpoint; disease progression was defined as initiation of FOLFOX salvage chemotherapy (used as a pragmatic proxy for disease recurrence). A second propensity score matching was performed directly between XELOX (n = 533) and TS-1 (n = 893) groups, yielding 490 matched pairs with well-balanced baseline characteristics. Multivariable Cox regression was adjusted for sex, age, comorbidities, and Charlson Comorbidity Index. TS-1 was associated with significantly better OS (adjusted HR 0.73, 95% CI 0.61–0.86; *p* < 0.001) and lower progression (adjusted HR 0.38, 95% CI 0.23–0.62; *p* < 0.001) versus XELOX; the corresponding 3-year OS was approximately 65.4% for TS-1 versus 56.8% for XELOX, and extrapolated 5-year OS approximately 50.2% versus 41.7%, respectively (note: these 5-year estimates are Kaplan–Meier projections beyond the mean follow-up of ~2.6 years and carry substantial uncertainty; they should be interpreted with caution). Benefits were confined to stage IIIA (OS HR 0.64, 95% CI 0.45–0.89; *p* = 0.009; interaction *p* = 0.006; progression HR 0.29, 95% CI 0.11–0.76; *p* = 0.011), with comparable outcomes in IIIB and IIIC. Adjuvant TS-1 monotherapy was associated with superior OS and lower disease progression versus XELOX in stage III gastric cancer, particularly in stage IIIA; these findings are hypothesis-generating and warrant confirmation in prospective randomized trials, whereas in stage IIIB/IIIC outcomes were comparable between the two regimens.

## 1. Introduction

Gastric cancer ranks as the fifth most common cancer and third leading cause of cancer-related death worldwide, with approximately 1.09 million new cases and 769,000 deaths in 2020 [1]; incidence and mortality are disproportionately high in East Asia, including Taiwan, where approximately 3500 new cases are diagnosed annually. Surgical resection with D2 lymphadenectomy is the standard curative-intent treatment in East Asian centers [2], yet even after complete (R0) resection, patients with pathological stage III disease retain a high relapse risk—5-year overall survival ranges approximately 40–60% depending on substage [3]—underscoring the need for effective adjuvant strategies.

Two landmark randomized controlled trials have defined the adjuvant chemotherapy landscape in Asia. The ACTS-GC trial demonstrated that adjuvant S-1 (tegafur/gimeracil/oteracil; TS-1) monotherapy significantly improved 3-year overall survival (80.1% vs. 70.1%) and relapse-free survival compared with surgery alone in Japanese patients with stage II and III gastric cancer after D2 gastrectomy [4,5]. Subsequently, the CLASSIC trial showed that adjuvant capecitabine plus oxaliplatin (XELOX/CAPOX) conferred significant improvements in 3-year disease-free survival (74% vs. 59%) and overall survival compared with surgery alone in patients from South Korea, China, and Taiwan after D2 resection for stage II through IIIB gastric cancer [6]. The 5-year follow-up of the CLASSIC trial further confirmed the durable benefit of XELOX, with an OS hazard ratio of 0.66 (95% CI 0.51–0.85; *p* = 0.0015) [7]. Critically, however, both trials enrolled mixed populations of stage II and III patients and were neither designed nor powered to assess outcomes independently within stage III substages (IIIA, IIIB, and IIIC). The optimal adjuvant regimen for each stage III substage therefore remains undefined by existing trial evidence.

Both TS-1 and XELOX are now included in major clinical practice guidelines in Asia as standard adjuvant options following D2 gastrectomy [3,8,9,10]. However, each regimen has its own strengths, creating clinical equipoise regarding optimal regimen selection. TS-1 monotherapy offers the advantages of oral administration, once-daily dosing, and a generally favorable tolerability profile in East Asian patients. In contrast, XELOX involves combination therapy with an intravenous oxaliplatin component, potentially offering greater cytotoxic potency but also carrying higher rates of cumulative peripheral neuropathy, myelosuppression, and infusion-related events. Head-to-head comparative effectiveness data specific to stage III patients—and particularly within individual substages—are therefore absent from existing trial evidence. Beyond the adjuvant setting, the rapidly evolving treatment paradigm for locally advanced gastric cancer is further illustrated by emerging evidence on neoadjuvant immunotherapy combined with chemotherapy: a recent retrospective cohort study reported encouraging outcomes with neoadjuvant PD-1 inhibitor combined with FLOT versus SOX in locally advanced gastric cancer, providing additional context for the contemporary therapeutic landscape [11].

Prior real-world studies have partially addressed this evidence gap. Kim et al. compared adjuvant TS-1 and XELOX in 206 Korean patients with stage II and III gastric cancer and found no significant overall survival difference, with only a trend toward TS-1 benefit in stage IIIC [12]. In a nationwide Korean claims-database cohort, Kim et al. also found no significant survival difference after propensity score matching; however, that analysis included stage II patients and lacked substage-specific comparisons within stage III [13]. To address these limitations, the present study utilized Taiwan’s National Health Insurance Research Database (NHIRD) linked with the Taiwan Cancer Registry–Long Form (TCR-LF) to compare adjuvant XELOX versus TS-1 monotherapy on overall survival and disease progression in an exclusively stage III cohort after curative D2 gastrectomy, with pre-specified substage analyses across IIIA, IIIB, and IIIC.

## 2. Methods

### 2.1. Data Source

This retrospective cohort study utilized NHIRD; 2000–2021, covering more than 99% of the Taiwanese population (approximately 23.5 million individuals) [14], linkage to the national death registry. Cancer staging information was obtained by linkage to the Taiwan Cancer Registry–Long Form (TCR-LF; 2007–2020), providing pathological TNM staging. This study was approved by the Research Ethics Committee of China Medical University and Hospital (CMUH113-REC2-143(CR-1)). Informed consent was waived due to the de-identified nature of the administrative data.

### 2.2. Study Population and Eligibility Criteria

**Figure 1:**
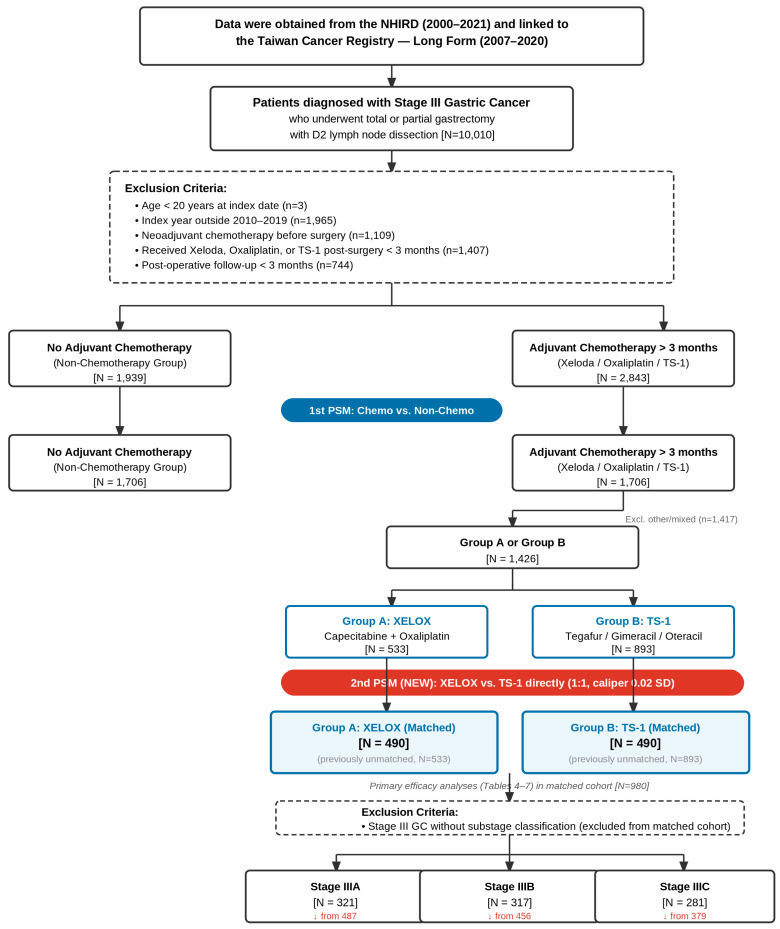
Study enrollment and patient flow diagram.

From the linked NHIRD–TCR database, we identified adult patients (aged ≥20 years) diagnosed with pathological stage III gastric cancer (ICD-9-CM 151.x/ICD-10-CM C16.x, with substage IIIA, IIIB, or IIIC confirmed by the TCR-LF) who underwent total or partial gastrectomy with D2 lymph node dissection between 1 January 2010, and 31 December 2019 (index period; N = 10,010). The index date was defined as the date of gastrectomy. All patients were followed until death, the occurrence of the progression endpoint, or December 31, 2020, whichever occurred first. The chemotherapy cohort comprised patients who received adjuvant chemotherapy, defined as the prescription of at least one of the following agents for at least 3 (i.e., ≥90 days, equivalent to ≥4 cycles for XELOX or the first three 28-day cycles for TS-1) consecutive months following surgery: capecitabine (Xeloda) and oxaliplatin, or TS-1. The intended full course duration was 6 months (8 cycles, 21-day cycle) for XELOX and 12 months for TS-1, in accordance with prevailing East Asian guidelines and the original CLASSIC and ACTS-GC trial protocols, respectively. Patients who received these agents for a cumulative duration of less than 3 months (i.e., <90 days) were excluded from the chemotherapy cohort (n = 1407). The following exclusion criteria were applied sequentially to the initial cohort (N = 10,010): (1) age younger than 20 years at the index date (n = 3); (2) index year outside the 2010–2019 study period (n = 1965); (3) receipt of neoadjuvant (preoperative) chemotherapy (n = 1109); (4) post-surgery use of capecitabine, oxaliplatin, or TS-1 for a cumulative duration of 3 months or less (n = 1407); and (5) post-operative follow-up duration of less than 3 months (n = 744). The overall study design and patient selection process are illustrated in Figure 1.

### 2.3. Chemotherapy Group Definitions

Among patients in the chemotherapy cohort (N = 2843), two mutually exclusive treatment groups were defined based on the specific adjuvant regimen received: (1) Group A (XELOX, n = 533): Patients who received both capecitabine (Xeloda) and oxaliplatin concurrently for more than 3 months postoperatively. (2) Group B (TS-1, n = 893): Patients who received TS-1 monotherapy for more than 3 months postoperatively, without concurrent oxaliplatin administration. A total of 1426 patients (XELOX, n = 533; TS-1, n = 893) formed the primary analytic cohort for the XELOX-versus-TS-1 comparison. The remaining 1417 patients received other or mixed regimens and were excluded from the primary analysis.

### 2.4. Outcome Definitions

The primary endpoint was overall survival (OS), defined as the time from the index date to all-cause death as ascertained from the national death registry linked to the NHIRD.

The secondary endpoint was disease progression, operationally defined as the initiation of FOLFOX-based (5-fluorouracil/leucovorin/oxaliplatin) salvage chemotherapy following at least 3 months of first-line adjuvant XELOX or TS-1. Progression-free survival (PFS) was defined as the time from the index date (date of surgery) to initiation of FOLFOX, the most commonly used salvage regimen for gastric cancer patients who experienced treatment failure following first-line adjuvant chemotherapy in Taiwan.

### 2.5. Propensity Score Matching

To minimize selection bias in the chemotherapy comparison, propensity scores were estimated using multivariable logistic regression incorporating the following covariates: sex, age group (20–49, 50–64, ≥65 years), type 2 diabetes mellitus, coronary artery disease, dyslipidemia, hepatitis B virus infection, hepatitis C virus infection, and Charlson Comorbidity Index (CCI) [15]; scored as 0–1, 2, or ≥3). PSM was performed at the cohort level (chemotherapy vs. non-chemotherapy) to first establish the comparability of patients who did and did not receive any adjuvant chemotherapy. A 1:1 nearest-neighbor matching algorithm with a caliper width of 0.02 standard deviations of the logit of the propensity score was applied [16]. Covariate balance was confirmed by standardized mean differences (SMD < 0.10).For the primary XELOX-versus-TS-1 comparison, among the 1426 chemotherapy patients (Group A: XELOX, n = 533; Group B: TS-1, n = 893), a second PSM was performed directly between the two regimen groups using the same covariates and 1:1 nearest-neighbor algorithm (caliper 0.02 SD), yielding 490 matched pairs. Covariate balance in the matched cohort was confirmed by SMD < 0.10 for all variables (Table 3). All primary efficacy analyses (Tables 4–7) were conducted in this regimen-matched cohort of 980 patients. Substage subgroup analyses were conducted within the regimen-matched cohort; patients without TCR-LF substage classification were excluded, yielding matched substage subgroups of Stage IIIA (N = 321), Stage IIIB (N = 317), and Stage IIIC (N = 281).

### 2.6. Statistical Analysis

Continuous variables were expressed as means with standard deviations (SD) and compared using independent samples t-tests. Categorical variables were expressed as frequencies and percentages and compared using chi-square tests. Cox proportional hazards regression models were used to estimate crude and adjusted hazard ratios (HR) with 95% confidence intervals (CI). Adjusted models incorporated sex, age, all comorbidities, and CCI as covariates. Kaplan-Meier (KM) survival curves were generated for OS and PFS with group comparisons by log-rank test. Subgroup analyses were stratified by pathological substage (IIIA, IIIB, IIIC). Formal tests for interaction between treatment group and substage were performed using a likelihood ratio test comparing Cox models with and without a treatment-by-substage interaction term; interaction *p*-values are reported alongside substage-specific HRs. The proportional hazards assumption was assessed using scaled Schoenfeld residuals. All analyses were performed using SAS version 9.4 (SAS Institute Inc., Cary, NC, USA). Statistical significance was defined as a two-tailed *p*-value < 0.05.

## 3. Results

### Baseline Characteristics After Propensity Score Matching

**Figure 2:**
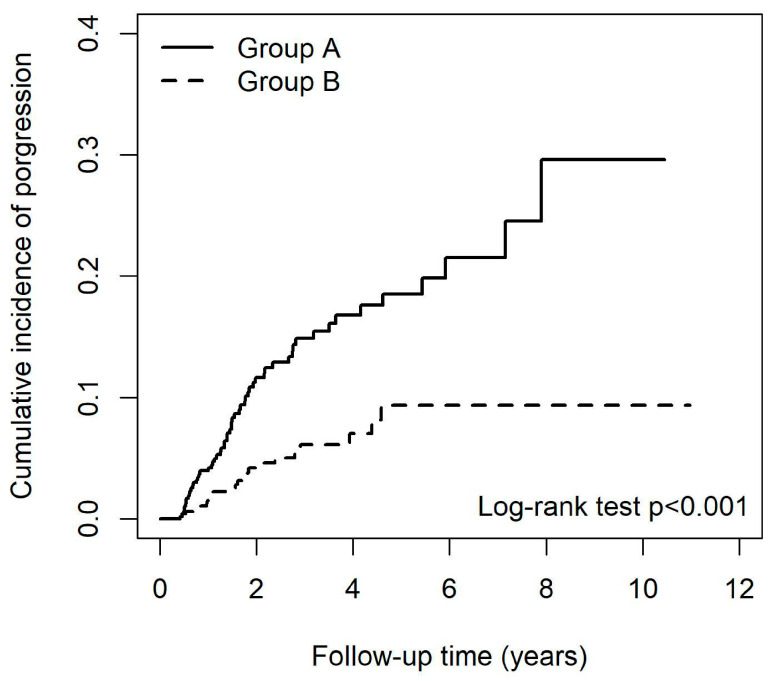
Kaplan-Meier curves for progression-free survival (PFS) in Group A (XELOX) versus Group B (TS-1 monotherapy). Progression was defined as initiation of FOLFOX-based salvage chemotherapy after at least 3 months of first-line adjuvant treatment. The *x*-axis represents follow-up time in years; the *y*-axis represents the probability of remaining progression-free. Log-rank *p* < 0.001. XELOX: capecitabine plus oxaliplatin; TS-1: tegafur/gimeracil/oteracil; FOLFOX: 5-fluorouracil, leucovorin, and oxaliplatin.

After 1:1 PSM, the chemotherapy and non-chemotherapy cohorts each comprised 1706 patients. The two cohorts were well-balanced across all baseline covariates (Table 1; all *p*-values were >0.04 after matching, with the exception of coronary artery disease [*p* = 0.045]). Mean age was 68.38 ± 12.05 years in the non-chemotherapy group and 68.37 ± 11.87 years in the chemotherapy group (*p* = 0.981). Male patients comprised 61.0% and 61.5% of the non-chemotherapy and chemotherapy cohorts, respectively (*p* = 0.752). CCI distribution was similarly comparable between groups (*p* = 0.168). Among chemotherapy recipients, 54.5% received capecitabine, 53.3% received oxaliplatin, and 69.1% received TS-1, with certain patients receiving combination regimens. Mean follow-up for OS analysis was 2.63 ± 2.31 years in the non-chemotherapy cohort and 2.64 ± 2.17 years in the chemotherapy cohort (*p* = 0.828). As shown in Table 2, in the PSM cohort, 876 deaths were observed in the non-chemotherapy group (IR 195.39 per 1000 PY) and 1012 deaths in the chemotherapy group (IR 224.31 per 1000 PY). Among covariates, older age (≥65 years: adjusted HR 1.76, 95% CI 1.43–2.17, *p* < 0.001) and male sex (adjusted HR 1.15, 95% CI 1.04–1.26, *p* = 0.005) were independently associated with higher mortality. Dyslipidemia (adjusted HR 0.87, 95% CI 0.78–0.96, *p* = 0.006) and hepatitis B infection (adjusted HR 0.80, 95% CI 0.67–0.96, *p* = 0.014) were associated with lower mortality risk in adjusted analyses. After regimen-level PSM (490 matched pairs), Group A (XELOX) contributed 306 deaths over 1339.5 person-years (IR 228.4 per 1000 PY), while Group B (TS-1 monotherapy) contributed 248 deaths over 1405.9 person-years (IR 176.4 per 1000 PY). Baseline characteristics of the regimen-matched XELOX and TS-1 monotherapy cohorts are summarized in Table 3, showing generally well-balanced covariates after propensity score matching. In unadjusted analysis, TS-1 was associated with significantly lower mortality (crude HR 0.77, 95% CI 0.66–0.92; *p* = 0.003). After adjustment for sex, age, comorbidities, and CCI, TS-1 was significantly associated with better OS (adjusted HR 0.73, 95% CI 0.61–0.86; *p* < 0.001; Table 4), representing a 27% reduction in the risk of death compared with XELOX. Kaplan-Meier survival curves showed diverging trajectories favoring TS-1 over the entire follow-up period (log-rank *p* < 0.001). With respect to disease progression (defined as transition to FOLFOX-based salvage chemotherapy), Group A experienced 58 progression events over 1287.6 person-years (IR 45.05 per 1000 PY), compared with only 23 progression events over 1392.1 person-years in Group B (IR 16.52 per 1000 PY). TS-1 monotherapy was associated with substantially lower risk of progression in both crude (HR 0.37, 95% CI 0.23–0.60; *p* < 0.001) and adjusted analyses (HR 0.38, 95% CI 0.23–0.62; *p* < 0.001; Table 5), corresponding to a 62% reduction in the risk of disease progression compared with XELOX. Kaplan-Meier PFS curves showed significant and early separation favoring TS-1 (Figure 2; log-rank *p* < 0.001). Stage-stratified analyses of OS revealed significant heterogeneity in the treatment effect of TS-1 across substages (formal interaction *p* = 0.006; Table 6). In Stage IIIA patients (N = 321), the adjusted HR for death with TS-1 versus XELOX was 0.64 (95% CI 0.45–0.89; *p* = 0.009), indicating a statistically significant 36% reduction in mortality risk favoring TS-1. In contrast, no statistically significant OS difference was observed in Stage IIIB patients (N = 317; adjusted HR 0.86, 95% CI 0.64–1.15; *p* = 0.307) or Stage IIIC patients (N = 281; adjusted HR 1.09, 95% CI 0.80–1.48; *p* = 0.583). The incidence rate of death progressively increased across substages in both groups, confirming the prognostic relevance of substage classification. In stage-stratified progression analyses (Table 7; formal interaction *p* = 0.217 for treatment-by-substage), TS-1 significantly reduced the risk of disease progression versus XELOX in Stage IIIA (adjusted HR 0.29, 95% CI 0.11–0.76; *p* = 0.011). In Stage IIIB, no significant reduction was observed (adjusted HR 0.61, 95% CI 0.26–1.38; *p* = 0.233). Similarly, in Stage IIIC, no significant progression benefit was found (adjusted HR 0.63, 95% CI 0.25–1.61; *p* = 0.335). The non-significant interaction *p*-value (*p* = 0.217) indicates that while point estimates numerically favor TS-1 across substages, statistically differential treatment effects by substage were not established for the progression endpoint.

## 4. Discussion

This nationwide, population-based cohort study using Taiwan’s NHIRD is among the largest real-world analyses to directly compare adjuvant XELOX and TS-1 monotherapy in stage III gastric cancer patients following curative D2 gastrectomy. Unlike Japan, where TS-1 is the dominant adjuvant regimen, or South Korea, where XELOX was widely adopted following the CLASSIC trial, Taiwan employs both regimens in routine clinical practice—creating an ecologically balanced prescription environment uniquely suited for a genuine head-to-head effectiveness comparison. The principal finding is that TS-1 monotherapy was associated with significantly better OS (adjusted HR 0.73; *p* < 0.001) and substantially lower disease progression (adjusted HR 0.38; *p* < 0.001) compared with XELOX in the propensity-matched cohort. A formal test for treatment-by-substage interaction confirmed significant heterogeneity in the OS benefit (interaction *p* = 0.006), with the survival benefit of TS-1 most pronounced and statistically significant in stage IIIA disease, whereas outcomes were comparable between the two regimens in stage IIIB and IIIC.

The current findings are consistent with the established efficacy of TS-1 demonstrated in the ACTS-GC trial, where adjuvant S-1 significantly improved OS in Japanese patients with stage II/III gastric cancer after D2 gastrectomy (5-year OS: 71.7% vs. 61.1% with surgery alone) [4]. While ACTS-GC was predominantly Japanese, pharmacogenomic data suggest that Taiwanese and other East Asian populations share similar CYP2A6 and dihydropyrimidine dehydrogenase (DPD) enzyme activity profiles that favor TS-1 tolerability and efficacy compared with Western populations [17]. This biological commonality may partly explain why TS-1 demonstrated superiority over XELOX in our Taiwanese cohort. Conversely, owing to these pharmacogenomic differences—including a higher prevalence of CYP2A6 polymorphisms causing greater fluoropyrimidine-related gastrointestinal toxicity in non-East Asian patients—the present findings may not generalize directly to Western populations and should be interpreted within the East Asian clinical context. The CLASSIC trial, which established XELOX as a standard adjuvant option in East Asia, demonstrated significant OS benefit over surgery alone, but did not include a direct TS-1 comparison arm [6]. The absence of a head-to-head comparison in the controlled trial setting has created ongoing uncertainty in clinical practice. Our real-world data suggest that, in the Taiwanese context, TS-1 may be the superior choice, at least for certain stage subgroups. It is important to note that both XELOX and TS-1 demonstrate efficacy relative to observation; the present analysis instead addresses the comparative question of which regimen performs better in real-world practice.

More recently, the JACCRO GC-07 trial demonstrated that adjuvant S-1 plus docetaxel significantly improved relapse-free survival and overall survival compared with S-1 alone in stage III gastric cancer following D2 gastrectomy, with 5-year OS of 67.9% versus 60.3% (HR 0.752; *p* = 0.006) [18,19]. This landmark finding has positioned S-1 plus docetaxel as a new standard of care in Japan for fit patients with stage III disease. However, the addition of docetaxel substantially increased treatment-related toxicity: grade 3 or higher adverse events occurred in 58% of patients in the combination arm versus 42% in the S-1-alone arm, driven by markedly elevated rates of neutropenia (39.2% vs. 16.4%), leukopenia (22.4% vs. 2.7%), and febrile neutropenia (5.7% vs. 0.4%) [18]. For patients who are elderly, have reduced performance status, or present with significant comorbidities following major surgery—a population commonly encountered in real-world clinical practice in Taiwan—this intensified regimen may be poorly tolerated or contraindicated. In this context, TS-1 monotherapy retains critical clinical importance as an effective and better-tolerated adjuvant option, as supported by the present study. Of note, docetaxel-based adjuvant regimens are not yet routinely reimbursed for stage III gastric cancer in Taiwan; in our cohort, patients who received any docetaxel-containing combination were captured under the “other or mixed regimens” category (n = 1417) and excluded from the primary XELOX-versus-TS-1 comparison, so contamination of either analytic group by S-1/docetaxel use is not expected. The XELOX–TS-1 comparison therefore remains the dominant clinical equipoise in Taiwan, where docetaxel-based adjuvant regimens are not yet routinely adopted outside Japan.

The heterogeneity of TS-1 benefit across stage IIIA versus IIIB/IIIC is a clinically important and biologically plausible finding. Stage IIIA represents an intermediate-risk category (T4aN0M0, T3N1M0, or T2N2M0) where tumor dissemination is relatively limited and the disease may be more amenable to the sustained, steady-state fluoropyrimidine exposure achievable with once-daily oral TS-1. In this setting, the tolerability advantage of TS-1—with lower rates of peripheral neuropathy, myelosuppression, and treatment discontinuation compared with XELOX—may translate into more consistent drug delivery and superior efficacy. In contrast, for stage IIIB and IIIC patients, who carry heavier nodal burden and higher risk of micrometastatic dissemination, the aggressive tumor biology may require more cytotoxic combination regimens, potentially explaining the convergence of outcomes between XELOX and TS-1 in these subgroups. An alternative interpretation is that stage IIIC patients in real-world practice may be less able to tolerate or complete full courses of either regimen, thereby attenuating differential efficacy. The finding that neither regimen shows a clear OS advantage in stage IIIB/IIIC highlights an unmet clinical need for more effective adjuvant strategies in higher-stage disease, such as immunotherapy integration or intensified combination regimens [20,21]. Importantly, to our knowledge no prior real-world study has directly compared TS-1 and XELOX across all three pathological substages (IIIA, IIIB, and IIIC) independently in the same cohort. This granular substage-level analysis represents a major contribution of the present study, translating the broad stage III classification into actionable guidance for individualized adjuvant regimen selection.

Several prior real-world studies have addressed the comparative efficacy of adjuvant S-1 and XELOX, though none has provided the substage-level resolution achieved in the present analysis. Kim and colleagues reported a retrospective Korean cohort of 206 patients with stage III gastric cancer after D2 gastrectomy, finding numerically higher 3-year OS with S-1 (75.6%) than XELOX (69.6%), without statistical significance; a non-significant trend favoring XELOX was noted specifically in stage IIIC (HR 0.50; *p* = 0.075) [12]. This aligns with our finding of comparable outcomes between the two regimens in stage IIIC, and the lack of statistical significance in that Korean analysis may be attributed to limited sample size. A larger nationwide population-based Korean cohort study using claims database data similarly concluded that S-1 was non-inferior or superior to XELOX following curative gastrectomy, consistent with the direction of our results [13]. In a separate Korean multicenter real-world analysis restricted to stage III disease, Kim et al. also reported no significant survival difference between adjuvant S-1 and XELOX; however, that analysis similarly lacked substage-level comparisons within stage III []. Taken together, available real-world data from East Asian populations consistently support TS-1 as at least equivalent to XELOX in the adjuvant setting for stage III gastric cancer, and potentially superior—particularly for stage IIIA disease—in larger, propensity-matched analyses such as the present study. Additionally, the present study uniquely extends this body of evidence in three respects. First, it provides a substantially larger directly propensity-matched regimen comparison (490 matched pairs), drawn from a near-complete national registry covering over 99% of the Taiwanese population. Second, it delivers the first prospectively planned substage-stratified comparison across IIIA, IIIB, and IIIC within the same cohort, with formal interaction testing confirming substage heterogeneity for OS (*p* = 0.006). Third, it is conducted in Taiwan—a setting where both XELOX and TS-1 are actively prescribed, unlike Japan (TS-1 dominant) or Korea (XELOX dominant after CLASSIC)—thereby generating the most ecologically balanced and informative real-world head-to-head evidence currently available for this clinical question.

The substantially lower rate of disease progression in Group B (TS-1; IR 16.52/1000 PY) compared with Group A (XELOX; IR 45.05/1000 PY), corresponding to an adjusted HR of 0.38, is a notable finding that corroborates the OS benefit of TS-1. The substantially lower progression rate suggests that TS-1 more effectively maintains disease control during the adjuvant period, thereby delaying or preventing the need for second-line FOLFOX-based salvage therapy. From a practical standpoint, delaying progression preserves quality of life and maintains options for subsequent lines of therapy, which is particularly important given the relatively limited therapeutic landscape in second-line gastric cancer. Our operational definition of progression—transition to FOLFOX—is a pragmatic claims-based surrogate that, while imperfect, captures the most clinically relevant treatment decision in the context of adjuvant failure. We acknowledge that defining progression by initiation of FOLFOX depends on a treatment decision rather than direct radiologic or pathologic confirmation of recurrence and is therefore subject to misclassification bias. Importantly, patients who received XELOX (containing oxaliplatin) may be less likely to receive FOLFOX (also oxaliplatin-based) as second-line therapy due to concerns about cumulative neurotoxicity or presumed oxaliplatin resistance, potentially inflating the apparent progression rate in the XELOX group and creating a circular bias. This is a recognized limitation of FOLFOX-initiation as a surrogate endpoint in XELOX-treated patients. In Taiwan’s NHIRD context, FOLFOX is the most frequently prescribed second-line regimen for gastric cancer patients failing first-line adjuvant fluoropyrimidine-based therapy, and prior internal audits suggest that the majority of patients with documented disease progression in stage III gastric cancer go on to receive FOLFOX-based salvage chemotherapy, supporting reasonable sensitivity of this surrogate. Nonetheless, some patients with progressive disease may instead receive best supportive care, alternative second-line regimens (e.g., ramucirumab–paclitaxel), or no further systemic therapy, which would be missed by our definition and may underestimate true progression rates. Because such non-FOLFOX salvage approaches are unlikely to be differentially distributed between XELOX and TS-1 recipients, the resulting misclassification is expected to be largely non-differential and to bias the comparative HR toward the null. Future studies incorporating direct radiologic recurrence end points are warranted to confirm the magnitude of the progression benefit. Notably, patients treated with XELOX may have a higher propensity to receive FOLFOX as a recognized second-line option given that both share the oxaliplatin backbone; however, this would likely underestimate the progression rate in Group A and bias results toward the null, suggesting the true progression benefit of TS-1 may be even greater than observed.

Several limitations of the present study require acknowledgment. First, as with all analyses utilizing administrative claims data, the NHIRD lacks granular clinical information such as tumor histology, tumor differentiation/grading, Lauren histological subtype, number of positive lymph nodes, nutritional status (e.g., albumin, body-mass index, prognostic nutritional index), HER2 status, microsatellite instability (MSI), performance status, surgical margin status, perioperative complications, and lymphovascular invasion—all of which influence prognosis and treatment selection. Because these oncologically relevant variables could not be entered into the propensity score model or as additional covariates in the multivariable Cox model, residual confounding from such factors cannot be fully excluded and could meaningfully affect the observed survival differences. In real-world Taiwanese practice, however, XELOX is more often selected for younger and fitter patients with adequate hematologic reserve and good performance status, while TS-1 is more often selected for older patients or those with comorbidities and limited tolerance of intravenous oxaliplatin; if anything, this prescribing pattern would have biased our results against TS-1 (i.e., better baseline prognosis in the XELOX group) and would render the observed TS-1 benefit even more conservative—strengthening rather than weakening the conclusions. Future prospective or registry-linked studies that explicitly capture pathological grading, lymph node ratio, and nutritional indices as confounders are warranted. Importantly, the NHIRD does not capture ECOG performance status, one of the most critical determinants of treatment selection and prognosis in gastric cancer. Patients with poorer performance status are more likely to receive TS-1—an oral, once-daily regimen with favorable tolerability—rather than XELOX, which is associated with peripheral neuropathy and pronounced hematologic toxicity, thereby introducing potential confounding by indication. Additionally, detailed toxicity profiles, dose reductions, and treatment modifications are not recorded in administrative claims data; patients who required early dose reductions or premature discontinuation due to toxicity are indistinguishable from those who completed the planned course, precluding any dose–response or adherence analysis. Furthermore, the specific pattern of tumor recurrence—locoregional, peritoneal, or distant hematogenous—cannot be ascertained from ICD codes alone, limiting mechanistic interpretation of the progression endpoint. Second, our surrogate endpoint for disease progression (FOLFOX initiation) may not capture all instances of disease recurrence, particularly if patients with progressive disease received best supportive care, alternative salvage regimens (e.g., ramucirumab–paclitaxel), or no further systemic therapy. A particularly important source of directional bias deserves explicit discussion: patients who received XELOX as adjuvant therapy carry prior oxaliplatin exposure. Upon disease recurrence, clinicians may preferentially avoid re-exposing these patients to FOLFOX—which also contains oxaliplatin—due to concerns about cumulative peripheral neurotoxicity or oxaliplatin resistance. If FOLFOX is systematically less likely to be prescribed to XELOX-pretreated patients at recurrence, the observed FOLFOX-initiation rate in Group A would be artificially suppressed, making the XELOX arm appear to have a lower apparent progression rate than it actually does. Critically, this directional bias operates against the observed superiority of TS-1: the true recurrence rate in the XELOX group may be even higher than our surrogate captures. The fact that XELOX nonetheless demonstrates a significantly higher FOLFOX-initiation rate (IR 45.05 vs. 16.52 per 1000 PY; adjusted HR 0.38, 95% CI 0.23–0.62; *p* < 0.001) indicates that the observed progression benefit of TS-1 is a conservative underestimate, strengthening rather than undermining the conclusion of superior disease control with TS-1. Third, dosing information, treatment adherence, cycle completion rates, and dose intensity are unavailable in the NHIRD, precluding analysis of dose-response relationships. Fourth, the retrospective design spanning 2008–2020 encompasses a period during which clinical guidelines and practice patterns evolved, potentially introducing temporal heterogeneity. Fifth, while the PSM effectively balanced observable characteristics, unmeasured confounders including surgeon experience, hospital volume, and patients’ socioeconomic determinants of health may have influenced both treatment selection and outcomes. Sixth, the median follow-up duration of approximately 2.6 years is relatively short for an OS analysis in stage III gastric cancer, where late recurrences and deaths beyond 5 years are not uncommon, and our survival data should therefore be considered an interim assessment of intermediate-term outcomes; longer follow-up will be needed to confirm whether the OS advantage of TS-1 over XELOX is sustained. Seventh, despite the direct PSM performed between XELOX and TS-1 groups (490 matched pairs), unmeasured confounders not captured in the propensity score model—including tumor grade, lymph node ratio, HER2/MSI status, performance status, and nutritional indices—cannot be fully excluded and may influence results. This study is therefore best interpreted as hypothesis-generating observational evidence rather than definitive evidence to change clinical practice. Despite these limitations, the large sample size, population-based design with near-complete national coverage, consistent findings across both OS and progression endpoints, and coherent stage-specific subgroup patterns collectively support the validity and clinical relevance of our results.

## 5. Conclusions

In this nationwide, propensity score–matched, real-world cohort study of stage III gastric cancer patients after D2 gastrectomy in Taiwan, adjuvant TS-1 monotherapy was associated with significantly better overall survival (adjusted HR 0.73; *p* < 0.001) and substantially lower disease progression (adjusted HR 0.38; *p* < 0.001) compared with XELOX in 490 directly matched pairs. A formal interaction test confirmed significant treatment heterogeneity by substage for OS (*p* = 0.006); the survival and progression benefits were most pronounced and statistically significant in stage IIIA patients (OS adjusted HR 0.64; *p* = 0.009; PFS adjusted HR 0.29; *p* = 0.011), while outcomes were comparable in stage IIIB and IIIC. These findings are hypothesis-generating and suggest that TS-1 monotherapy may be a favorable adjuvant option particularly for stage IIIA disease in East Asian patients, whereas outcomes in stage IIIB/IIIC appear comparable between regimens. Given the observational design, unmeasured confounders, surrogate progression endpoint, and relatively short follow-up, these findings should not be used to change clinical practice without confirmation by prospective randomized trials. Given the pharmacogenomic and tolerability differences between East Asian and non-East Asian populations, these results should not be extrapolated uncritically to Western patients. Prospective randomized trials directly comparing XELOX and TS-1 in stage-defined subgroups are warranted to confirm these findings and guide evidence-based regimen selection.

## Figures and Tables

**Table 1:** Baseline characteristics of chemotherapy and non-chemotherapy cohorts after propensity score matching.

Variables	No Chemo n (%)		Chemo n (%)		*p*-Value
	N = 1706		N = 1706		
Chemotherapy agents					
Capecitabine (Xeloda)	—		930	54.51	
Oxaliplatin	—		909	53.28	
TS-1	—		1178	69.05	
Sex					0.752
Female	665	38.98	656	38.45	
Male	1041	61.02	1050	61.55	
Age (years)					0.955
20–49	121	7.09	117	6.86	
50–64	504	29.54	509	29.84	
≥65	1081	63.36	1080	63.31	
Mean ± SD ^a^	68.38	±12.05	68.37	±11.87	0.981
Comorbidities					
Type 2 diabetes mellitus	597	34.99	555	32.53	0.128
Coronary artery disease	593	34.76	538	31.54	0.045
Dyslipidemia	788	46.19	801	46.95	0.655
Hepatitis B virus	152	8.91	159	9.32	0.677
Hepatitis C virus	67	3.93	59	3.46	0.468
Charlson Comorbidity Index					0.168
0–1	38	2.23	26	1.52	
2	750	43.96	788	46.19	
≥3	918	53.81	892	52.29	
**Follow-up duration (years)**					
Death, Mean ± SD ^a^	2.63	±2.31	2.64	±2.17	0.828
Progression, Mean ± SD ^a^	2.61	±2.31	2.57	±2.17	0.598

^a^ T-test. All other comparisons by chi-square test. SD: standard deviation; Chemo: chemotherapy; —: not applicable (non-chemotherapy group). Note: Patients may have received more than one chemotherapy agent; percentages for individual agents reflect proportions within the chemotherapy cohort.

**Table 2:** Hazard ratio and 95% confidence intervals of death: chemotherapy versus non-chemotherapy cohort (after propensity score matching).

Variables	n	Person-Years	IR ^1^	Crude HR	(95% CI)	*p*-Value	Adjusted HR ^2^	(95% CI)	*p*-Value
Sex									
Female	673	3578.20	188.08	1.00	(reference)	—	1.00	(reference)	—
Male	1215	5416.70	224.31	1.19	(1.08, 1.31) ***	<0.001	1.15	(1.04, 1.26) **	0.005
Age (years)									
20–49	98	737.53	132.88	1.00	(reference)	—	1.00	(reference)	—
50–64	465	2960.89	157.05	1.18	(0.95, 1.47)	0.130	1.17	(0.94, 1.45)	0.169
≥65	1325	5296.49	250.17	1.82	(1.48, 2.24) ***	<0.001	1.76	(1.43, 2.17) ***	<0.001
Type 2 diabetes mellitus									
No	1215	6146.79	197.66	1.00	(reference)	—	1.00	(reference)	—
Yes	673	2848.11	236.30	1.16	(1.06, 1.28) **	0.002	1.11	(0.99, 1.24)	0.067
Coronary artery disease									
No	1224	6091.43	200.94	1.00	(reference)	—	1.00	(reference)	—
Yes	664	2903.47	228.69	1.13	(1.03, 1.24) *	0.012	0.95	(0.86, 1.05)	0.307
Dyslipidemia									
No	1029	4879.00	210.90	1.00	(reference)	—	1.00	(reference)	—
Yes	859	4115.91	208.70	0.97	(0.89, 1.06)	0.548	0.87	(0.78, 0.96) **	0.006
Hepatitis B virus									
No	1751	8105.79	216.02	1.00	(reference)	—	1.00	(reference)	—
Yes	137	889.12	154.08	0.72	(0.60, 0.85) ***	<0.001	0.80	(0.67, 0.96) *	0.014
Hepatitis C virus									
No	1815	8678.83	209.13	1.00	(reference)	—	1.00	(reference)	—
Yes	73	316.07	230.96	1.08	(0.86, 1.37)	0.515	1.05	(0.83, 1.33)	0.682

^1^ IR: incidence rate per 1000 person-years. ^2^ Adjusted for sex, age, and all listed comorbidities. CI: confidence interval; HR: hazard ratio. * *p* < 0.05; ** *p* < 0.01; *** *p* < 0.001.

**Table 3:** Baseline characteristics of Group A (XELOX) and Group B (TS-1 monotherapy) after propensity score matching.

Variables	Group A: XELOX (N = 490)		Group B: TS-1 Alone (N = 490)		*p*-Value
	n	%	n	%	
Sex					
Female	170	34.69	185	37.76	0.319
Male	320	65.31	305	62.24	
Age					
20–49	38	7.76	37	7.55	0.182
50–64	209	42.65	182	37.14	
≥65	243	49.59	271	55.31	
^a^ Mean (SD)	64.4 (10.32)		65.83 (11.17)		0.038
Comorbidities					
Type 2 diabetes mellitus	153	31.22	168	34.29	0.307
Coronary artery disease	153	31.22	139	28.37	0.328
Dyslipidemia	230	46.94	220	44.90	0.522
Hepatitis B virus	53	10.82	46	9.39	0.458
Hepatitis C virus	16	3.27	15	3.06	0.855
CCI					
0–2	258	52.65	222	45.31	0.021
≥3	232	47.35	268	54.69	
Follow-up duration (years)					
Death, ^a^ Mean (SD)	2.73 (2.17)		2.87 (2.29)		0.342
Progression, ^a^ Mean (SD)	2.63 (2.14)		2.84 (2.29)		0.133

Chi-square; ^a^ T-test. SD: standard deviation; CCI: Charlson Comorbidity Index.

**Table 4:** Hazard ratio and 95% confidence intervals of overall survival: Group A (XELOX) versus Group B (TS-1 monotherapy).

				Crude			Adjusted ^1^		
Variables	n	PY	IR	HR	(95% CI)	*p*-Value	HR	(95% CI)	*p*-Value
Group A: Xeloda + Oxaliplatin	306	1339.50	228.443	1	(reference)	-	1	(reference)	-
Group B: TS-1 alone	248	1405.91	176.399	0.77	(0.66, 0.92) **	0.003	0.73	(0.61, 0.86) ***	<0.001
Sex									
Female	193	1022.55	188.745	1	(reference)	-	1	(reference)	-
Male	361	1722.86	209.535	1.11	(0.93, 1.32)	0.242	1.02	(0.85, 1.22)	0.815
Age									
20–49	28	201.59	138.898	1	(reference)	-	1	(reference)	-
50–64	194	1094.19	177.300	1.35	(0.91, 2.00)	0.142	1.32	(0.89, 1.97)	0.17
≥65	332	1449.63	229.024	1.81	(1.23, 2.66) **	0.003	1.82	(1.22, 2.71) **	0.003
Comorbidities									
Type 2 diabetes mellitus									
No	364	1871.43	194.504	1	(reference)	-	1	(reference)	-
Yes	190	873.98	217.396	1.1	(0.93, 1.31)	0.274	1.08	(0.88, 1.32)	0.486
Coronary artery disease									
No	380	1864.30	203.830	1	(reference)	-	1	(reference)	-
Yes	174	881.10	197.480	0.99	(0.83, 1.19)	0.933	0.85	(0.70, 1.03)	0.093
Dyslipidemia									
No	299	1491.59	200.458	1	(reference)	-	1	(reference)	-
Yes	255	1253.82	203.379	1	(0.84, 1.18)	0.975	0.92	(0.76, 1.11)	0.374
Hepatitis B virus									
No	508	2456.59	206.791	1	(reference)	-	1	(reference)	-
Yes	46	288.82	159.270	0.76	(0.56, 1.03)	0.076	0.86	(0.63, 1.16)	0.318
Hepatitis C virus									
No	534	2671.35	199.899	1	(reference)	-	1	(reference)	-
Yes	20	74.06	270.052	1.42	(0.91, 2.22)	0.121	1.38	(0.88, 2.17)	0.163
CCI									
0–2	266	1328.04	200.295	1	(reference)	-	1	(reference)	-
≥3	288	1417.37	203.194	1.02	(0.86, 1.21)	0.815	1.01	(0.84, 1.22)	0.912

^1^ IR: incidence rate per 1000 person-years. Adjusted for sex, age, comorbidities, and CCI. CI: confidence interval; HR: hazard ratio. ** *p* < 0.01; *** *p* < 0.001.

**Table 5:** Hazard ratio and 95% confidence intervals of disease progression: Group A (XELOX) versus Group B (TS-1 monotherapy).

				Crude			Adjusted ^1^		
Variables	n	PY	IR	HR	(95% CI)	*p*-Value	HR	(95% CI)	*p*-Value
Group A: Xeloda + Oxaliplatin	58	1287.56	45.047	1	(reference)	-	1	(reference)	-
Group B: TS-1 alone	23	1392.06	16.522	0.37	(0.23, 0.60) ***	<0.001	0.38	(0.23, 0.62) ***	<0.001
Sex									
Female	26	996.20	26.099	1	(reference)	-	1	(reference)	-
Male	55	1683.41	32.672	1.25	(0.78, 1.99)	0.355	1.28	(0.79, 2.08)	0.308
Age									
20–49	8	198.47	40.309	1	(reference)	-	1	(reference)	-
50–64	41	1058.72	38.726	1	(0.47, 2.14)	0.996	0.99	(0.46, 2.15)	0.984
≥65	32	1422.43	22.497	0.61	(0.28, 1.33)	0.213	0.67	(0.30, 1.53)	0.346
Comorbidities									
Type 2 diabetes mellitus									
No	59	1824.80	32.332	1	(reference)	-	1	(reference)	-
Yes	22	854.81	25.737	0.79	(0.48, 1.29)	0.339	0.75	(0.42, 1.33)	0.323
Coronary artery disease									
No	59	1818.98	32.436	1	(reference)	-	1	(reference)	-
Yes	22	860.63	25.563	0.82	(0.50, 1.33)	0.415	0.91	(0.53, 1.57)	0.745
Dyslipidemia									
No	46	1454.08	31.635	1	(reference)	-	1	(reference)	-
Yes	35	1225.54	28.559	0.89	(0.57, 1.38)	0.595	1.07	(0.64, 1.79)	0.804
Hepatitis B virus									
No	68	2403.82	28.288	1	(reference)	-	1	(reference)	-
Yes	13	275.79	47.137	1.64	(0.91, 2.97)	0.103	1.44	(0.79, 2.64)	0.237
Hepatitis C virus									
No	78	2606.43	29.926	1	(reference)	-	1	(reference)	-
Yes	3	73.18	40.993	1.46	(0.46, 4.64)	0.519	1.77	(0.54, 5.74)	0.344
CCI									
0–2	39	1298.21	30.041	1	(reference)	-	1	(reference)	-
≥3	42	1381.40	30.404	1.02	(0.66, 1.58)	0.925	1.16	(0.71, 1.88)	0.551

^1^ IR: incidence rate per 1000 person-years. Adjusted for sex, age, comorbidities, and CCI. Progression = initiation of FOLFOX-based salvage chemotherapy. CI: confidence interval; HR: hazard ratio. *** *p* < 0.001.

**Table 6:** Hazard ratio and 95% confidence intervals of overall survival with Group A (XELOX) and Group B (TS-1) in substage subgroups.

Subgroup	Group A: XELOX			Group B: TS-1 Alone			Crude			Adjusted ^1^			Interaction *p*
	n	PY	IR	n	PY	IR	HR	(95% CI)	*p*	HR	(95% CI)	*p*	
**Stage IIIA (N = 321)**	67	381.146	175.786	79	628.745	125.647	0.72	(0.52, 1.00) *	0.048	0.64	(0.45, 0.89) **	0.009	0.006
**Stage IIIB (N = 317)**	99	457.179	216.545	87	482.436	180.335	0.86	(0.65, 1.15)	0.324	0.86	(0.64, 1.15)	0.307	
**Stage IIIC (N = 281)**	128	471.890	271.250	75	226.359	331.332	1.21	(0.91, 1.60)	0.199	1.09	(0.80, 1.48)	0.583	

^1^ Adjusted for sex, age, comorbidities, and CCI. IR: incidence rate per 1000 person-years. * *p* < 0.05; ** *p* < 0.01.

**Table 7:** Hazard ratio and 95% confidence intervals of disease progression with Group A (XELOX) and Group B (TS-1) in substage subgroups.

Subgroup	Group A: XELOX			Group B: TS-1 Alone			Crude			Adjusted ^1^			Interaction *p*
	n	PY	IR	n	PY	IR	HR	(95% CI)	*p*	HR	(95% CI)	*p*	
**Stage IIIA**	13	362.871	35.825	7	624.763	11.204	0.32	(0.13, 0.79) *	0.014	0.29	(0.11, 0.76) *	0.011	0.217
**Stage IIIB**	16	445.034	35.952	9	477.339	18.855	0.53	(0.23, 1.21)	0.131	0.61	(0.26, 1.38)	0.233	
**Stage IIIC**	25	452.117	55.295	6	221.880	27.042	0.5	(0.20, 1.21)	0.125	0.63	(0.25, 1.61)	0.335	

^1^ Adjusted for sex, age, comorbidities, and CCI. Progression = initiation of FOLFOX-based salvage chemotherapy. IR: incidence rate per 1000 person-years. * *p* < 0.05.

## Data Availability

The data used in this study were derived from the Taiwan NHIRD. Due to legal and ethical restrictions imposed by the data provider, the data are not publicly available. Access to the data may be granted to qualified researchers upon reasonable request and with approval from the relevant authorities in Taiwan.
